# Effectiveness of Devices to Monitor Biofouling and Metals Deposition on Plumbing Materials Exposed to a Full-Scale Drinking Water Distribution System

**DOI:** 10.1371/journal.pone.0169140

**Published:** 2017-01-06

**Authors:** Maneesha P. Ginige, Scott Garbin, Jason Wylie, K. C. Bal Krishna

**Affiliations:** 1 CSIRO Land and Water, Wembley, Western Australia, Australia; 2 Water Corporation of Western Australia, Leederville, Western Australia, Australia; 3 School of Computing Engineering and Mathematics, Western Sydney University, Penrith, New South Wales, Australia; Institute of Materials Science, GERMANY

## Abstract

A Modified Robbins Device (MRD) was installed in a full-scale water distribution system to investigate biofouling and metal depositions on concrete, high-density polyethylene (HDPE) and stainless steel surfaces. Bulk water monitoring and a KIWA monitor (with glass media) were used to offline monitor biofilm development on pipe wall surfaces. Results indicated that adenosine triphosphate (ATP) and metal concentrations on coupons increased with time. However, bacterial diversities decreased. There was a positive correlation between increase of ATP and metal deposition on pipe surfaces of stainless steel and HDPE and no correlation was observed on concrete and glass surfaces. The shared bacterial diversity between bulk water and MRD was less than 20% and the diversity shared between the MRD and KIWA monitor was only 10%. The bacterial diversity on biofilm of plumbing material of MRD however, did not show a significant difference suggesting a lack of influence from plumbing material during early stage of biofilm development.

## Introduction

Drinking water comes into contact with different surfaces made from a variety of materials during its travel from source to consumer. Copper has been the preferred material for domestic plumbing due to its low cost and antimicrobial properties [1, 2]. The main distribution pipe networks, however, are often made up of cement mortar lined ductile cast iron or high-density polyethylene (HDPE). The material of choice is typically based on factors such as cost and durability [3]. In Western Australia, the distribution system contains a great deal of variability from design (above and below ground pipelines) to age of installation. In addition, variations in bulk water temperature, nutrients, hydraulic conditions, disinfectant residual and pipe material has provided a challenge for the maintenance of drinking water quality in the network. Colonization of biofilms on pipe walls is influenced by all of the factors mentioned above and it is often difficult to relate bacterial colonization on surfaces to a single determining factor [4].

From a public health perspective, there are significant pressures to suppress bacterial growth in distribution systems [5–9]. Corrosion, declining water quality, taste and odor, disinfectant decay, and an increased abundance of pathogens are some of the adverse consequences triggered by pipe wall biofilm in a distribution system [10, 11]. Even with a reduction of assimilable organic carbon (AOC) and maintenance of a disinfectant residual, a complete reduction of biofilms in distribution systems remain unrealistic [12]. One reason for this could be related to the type of pipe material and its characteristics [13]. Pipe material could have a major influence on the amount, rate and type of biofilm formation, the occurrence of pathogens and the effectiveness of the disinfectant used [13–15]. Hence, developing a better understanding of the biofouling properties of different pipe materials is critical to ensure safe delivery of drinking water to the consumer.

Currently, none of the materials selected for use in drinking water systems illustrates a clear resistance towards biofouling and results regarding the biofouling potential of pipe materials are inconsistent [16–20]. For example, Niquette et al. [17] revealed that plastic-based materials (Polyethylene (PE) and Polyvinyl chloride (PVC)) support less biofilm formation than iron, tarred steel and cement-based materials; whereas others showed no significant differences between biofouling of the stainless steel, PE, and PVC [14, 19]. Comparing PE and PVC pipes, Schwartz et al. [21] revealed that PE was able to support more biofilm than PVC and that PVC supported even less biofilm than stainless steel. Hallam et al. [16] ranked biofilm activity on pipe material in the order of glass (136 pg ATP cm^-2^) < cement (212 pg ATP cm^-2^) < PE (302 pg ATP cm^-2^) < PVC (509 pg ATP cm^-2^). Majority of these studies have been carried out in laboratory-scale and replicating natural environmental conditions (e.g. temperature variations), hydraulics (e.g. water pressure and flow variations etc.) and bulk water characteristics (e.g. disinfection residuals, salinity, pH, natural organic matter etc.) of full-scale distribution systems is paramount if laboratory-scale experiments are to reflect true biofouling potentials of different pipe materials. It is difficult to replicate conditions of full-scale distribution systems in laboratory-scale and this is perhaps the reason there are reports of inconsistent observations in past literature.

The Modified Robbins Device (MRD) is a well-established system suitable for direct monitoring of biofilm formation in drinking water distribution systems [22, 23]. It has been used in full-scale water distribution systems [24–26] and past studies confirm ability to facilitate undisturbed sampling of biofilm on a variety of surfaces exposed to natural conditions of a tubular flow system [23, 27, 28]. Additionally, the reproducibility of the MRD has been statistically demonstrated by Kerr et al. [29] suggesting that the use of MRD(s) to compare different materials or treatments is conducive.

Indirect methods such as assessment of (1) AOC [30], (2) biodegradable dissolved organic carbon [31], and (3) growth curves of planktonic bacteria in bulk water have also been explored to infer biofouling potentials in drinking water distribution systems. Having recognised the complexities around using chemical methods to assess biological stability of a distribution system, van der Kooij et al. [32] proposed a KIWA monitoring device (KIWA, Netherlands) to study biofilm formation (i.e. rate and extend of formation) in drinking water distribution systems. Additionally, use of this device has been further extended to investigate occurrences of discoloured water [7] and pathogenic organisms [33] in drinking water distribution systems. Unlike the MRD, this offline monitor is cost effective and is easy to install to facilitate routine biological and chemical assessment of drinking water distribution systems. However, maintenance of a high water flow rate (e.g. 4.6 L min^-1^ as proposed by van der Kooij et al. [32]) through the device results in wastage of a significant volume of water and this is a concern for most utilities.

For a drinking water utility, having an accurate understanding regarding biofouling of its pipe network is critical not only to ensure safe delivery of drinking water to its customers but also to manage material deterioration [34, 35]. While a MRD installed on a full-scale drinking water distribution system could accurately reflect the biofouling potential of pipe material, for continuous assessment of biofouling throughout networks, inexpensive offline monitoring devices (e.g. KIWA monitors) are essential. Hence, this study focused on using a MRD to explore early development of biofilm on pipe materials (specifically on concrete, HDPE and stainless steel) exposed to operational conditions (environmental, hydraulic and bulk water) of the Goldfields & Agricultural Water Supply System (G&AWSS) in Western Australia. A KIWA monitor was used as an offline device and was operated maintaining a reduced flow rate of water. Accordingly, the KIWA monitor was not exposed to all conditions of the full-scale distribution system and was operated to explore correlations between bacterial community composition and metals deposition on MRD and KIWA monitor. Additionally, with acknowledgement of the inability to monitor bacterial communities of a mature biofilm using bulk water, bulk water bacterial community composition was compared against pipe wall biofilms of different ages to examine whether community similarities exist during a particular age of a biofilm. The main objectives of the study were (1) to compare bacterial colonization and metals deposition on different pipe materials when exposed to conditions of a real distribution system; and (2) to compare bacterial community compositions and metals deposition on pipe wall biofilms of different ages with community compositions and metals concentrations of bulk water and KIWA monitor.

## Materials and Methods

### Biofilm monitoring setup on the full-scale distribution system

**Fig 1:**
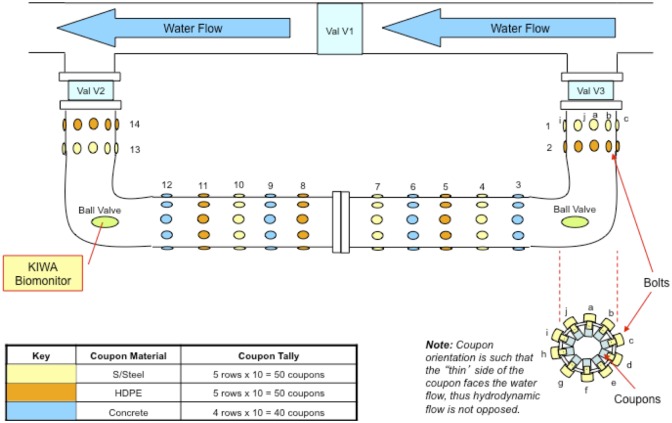
Schematic of MRD detailing the layout of coupons (pipe materials) and connection to the KIWA biofilm monitor.

A MRD (length: 349 cm and diameter: 16.9 cm) was installed in the G&AWSS in Western Australia that receives water from an integrated network (desalinated water, surface water and treated ground water). Chloramine is used as a disinfectant and due to temperature variations and higher retention times, some remote locations of the distribution network experience reduced bulk water disinfection residuals. The decay of chloramine along distribution systems is well demonstrated in past literature [36]. The MRD was installed at a location where chloramine residuals (measured as total chlorine) remained less than 0.2 mg L^-1^ to minimise the effect of disinfectant on biofilm growth and on microbial community composition. As shown in Fig 1, the MRD was placed in series as a by-pass to the main distribution line. Three valves (V1, V2 and V3) were installed to allow channelling of water through the MRD. Biofouling of different pipe materials (concrete, HDPE and stainless steel) was investigated by inserting coupons (4 cm x 1.5 cm) into the MRD. The coupons were orientated along the MRD as 14 rings (Fig 1). Each ring was composed of 10 coupons (labelled a to j) of a single pipe material. There were two opposing coupons at any given arrangement of the ring (e.g. coupon “a” and “f” opposed each other). One ball valve of the MRD was used to connect a KIWA monitor and other was used to release entrapped air from the MRD. The KIWA monitor and its operation are detailed in Ginige et al. [7]. Specifically, a low water flow rate of 0.28 L min^-1^ was maintained through the device. The Reynold's number, which reflects flow characteristics was calculated using Eq 1.

$$Re=\;\frac{\rho *V_{ag}*D}{\mu }$$ (1)

where, Re = Reynold's number

ρ = water density, kg/m^3^

V_ag_ = average water velocity, m/s

D = pipe diameter, m

μ = dynamic viscosity, N. s/m^2^

### Sampling of biofilm and bulk water

Bulk water and biofilm sampling (MRD and KIWA monitor) was carried out fortnightly during the first three months and monthly thereafter for another three months (spanning from end of Oct to end of Apr). During the first three months, the MRD coupons in rings 1 (stainless steel), 2 (HDPE), 3 (concrete), 9 (concrete), 10 (stainless steel) and 11 (HDPE) were sampled and during the latter 3 months coupons of rings 5 (stainless steel), 6 (HDPE), 7 (concrete), 12 (HDPE), 13 (concrete) and 14 (stainless steel) were sampled.

Prior to sampling, the water was diverted from the MRD by opening valve V1 and closing valves V2 and V3. Subsequently, the two opposing coupons of the relevant rings were removed one at a time so as to minimise drying of attached biofilm. Sampling of the attached biofilm was carried out by swabbing one side of the coupon (6.0 cm^2^). Swabbing was limited to 10 times and in between swabbing the swab was immersed into a sterile 25 mL Falcon tube containing 5 mL of autoclaved dechlorinated tap water to further moisten the swab and also to dislodge any swabbed biomass. The biofilm suspensions obtained from swabbing were transported and stored at 4°C.

Three glass rings of the KIWA monitor were sampled to analyse bacterial community compositions, ATP and metals (iron and manganese) deposition during a single sampling event and were placed inside two sterile 50 mL falcon tubes prior to transport and storage at 4°C. One falcon tube with two glass rings contained 10 mL of autoclaved dechlorinated tap water. The third glass ring contained in another falcon tube was used to analyse iron and manganese deposits. Additionally, 10 L of bulk water was also collected and stored in a sterile drum at 4°C from a sampling tap located near the MRD. The sampling tap was kept flowing for 5 min prior to collecting the bulk water.

### Sample processing and analysis

#### Processing of coupons, glass rings and bulk water

Biofilm suspensions (5 mL swabs) of respective MRD coupons and KIWA glass rings (in 10 mL of autoclaved dechlorinated tap water) were placed in an ultrasonic water bath (Bransonic 220, USA) for 3 min to dislodge attached biofilm. The suspensions were then decanted into new tubes and the biofilm suspension and glass rings were sonicated once more for 3 min in fresh autoclaved dechlorinated tap water (5 mL for swabs and 10 mL for rings). The corresponding suspensions of coupons and glass rings were pooled and an aliquot of each was subjected to an ATP analysis. The remainder of suspensions were filtered (0.22 μm Polycarbonate membrane filter) to concentrate detached biomass. Subsequently, the two retentates were resuspended separately in 500 μL of autoclaved dechlorinated tap water. An aliquot of each of the suspensions was used for metal analysis and the remainder was used to extract DNA.

Of the 10 L bulk water, 100 μL was used for ATP analysis and 50 mL was acidified using 10 μL of concentrated HNO_3_ for metals analysis. The remaining bulk water was then concentrated using a hollow fibre ultrafiltration system (HFUFS), fitted with a Hemoflow HF80S dialysis filter (Fresenius Medical Care, Lexington, MA, USA) as described by Hill et al. [37]. The concentrated samples were subsequently used to extract DNA.

#### Iron, manganese and ATP analysis

Ultra Trace Geoanalytical Laboratories, Australia, performed iron and manganese measurements on biofilm and in bulk water using Inductively Coupled Plasma Optical Emission Spectroscopy (ICP-OES). Quantitative estimation of iron and manganese was given precedence over other metals considering the influence these two metals have on discoloured water events [38]. ATP concentrations (in relative light units) were measured using the Promicol Biomass Detection Kit (Cat. # 360–0208) and Celsis Biocounter M1500 (Lumac, Netherlands). Iron, manganese and ATP were analysed in triplicate for each sample and additional details can be found in Ginige et al. [7]. ATP provides a quantitative estimation of viable microorganisms present in samples.

#### Bacterial community analysis using 454 sequencing

A total of 168 samples were pooled to make up 14 different trials (3 each for stainless steel, HDPE, concrete & bulk water and 2 for glass) to examine 3 biofilm ages of 0 to 1 month, 2 to 4 months and 5 to 6 months. The DNA of the 14 trials were extracted using the PowerSoil^®^ DNA Isolation Kit (MO BIO Laboratories, Inc., USA). The DNA extractions were then stored at -20°C prior to shipment to MR DNA (Molecular research LP, Texas, USA) for 454 pyrosequencing. Shipment was at room temperature on stabilising the DNA using DNA stable Plus (Biometrica, Diagnostic Technology). 454 pyrosequencing was performed as described previously [39]. In brief, HotStart Taq Plus Master Mix (Qiagen, CA, USA) was used for a single-step 30 cycle PCR amplification using 16S universal bacterial primers 27F (5′-AGRGTTTGATCMTGGCTCAG-3′) and 530R (5′-CCGCNGCNGCTGGCAC-3′). The thermocycler conditions used included an initial denaturing step at 94°C for 3 min followed by 28 cycles of denaturation at 94°C for 30 s; annealing at 53°C for 40 s; and an elongation at 72°C for 1 min; and a final elongation step at 72°C for 5 min. Amplicon products from different samples were mixed in equal concentrations, and purified using Agencourt Ampure beads (Agencourt Bioscience Corporation, MA, USA). Sequencing was carried out utilizing a Roche 454 FLX titanium instrument and reagents.

Post sequence processing was carried out using the QIIME (Quantitative Insights Into Microbial Ecology [40]) software package (http://www.qiime.org). Briefly, fasta, qual and mapping files were used as input for the split_libraries.py script with default arguments (except for the maximum sequence length, which was set at 600) to extract sequences relevant to this study. Subsequently, all sequences were grouped into operational taxonomic units (OTUs) using the pick_otus.py script in QIIME adopting the usearch method [41]. The sequence similarity threshold was set at 97% and the minimum cluster size was set at 1. The USEARCH sequence analysis method carried out clustering, chimera checking, denoising and also performed quality checks and filtering of de-multiplexed sequences [41]. A representative sequence from each OTU was selected and aligned against the Greengenes imputed core reference alignment using align_seqs.py script (with default alignment method PyNAST [42]). The gaps of the aligned sequence were then removed using script filter_alignment.py. A phylogenetic tree was constructed using the script make_phylogeny.py (with default settings Fast Tree). A taxonomy assignment (with script assign_taxonomy.py) was performed using the Ribosomal Database Project classifier and Greengenes OTUs data set (minimum confidence level 0.8). Finally, a rarefaction curve, bacterial species richness (Chao1), phylogenetic diversity (PD) and bacterial diversity (Shannon index) for each sample were derived using the alpha_rarefaction.py script. The unprocessed DNA sequences of this study were deposited (accession number 4543357.3) in MG-RAST [43].

### Statistical analysis

To explore the impact of pipe material on chemical and biological fouling, a one-way between-groups analysis of variance was conducted using SPSS version 13 (SPSS, Chicago). The samples were divided into 3 groups according to biofilm age (0 to1 month, 2 to 4 months and 5 to 6 months) to facilitate this analysis. The percentage abundance of sequences in each OTU (calculated based on total sequences of each sample) was compared using program PAST [44] to assess bacterial similarities between samples. Specifically a hierarchical cluster analysis was performed using the Bray-Curtis similarity measure at a bootstrap of N = 100. Further, a principle coordinate analysis (PCoA) was performed using the Bray-Curtis similarity index.

## Results and Discussion

### Biological and chemical fouling of pipe material and KIWA monitor

**Fig 2:**
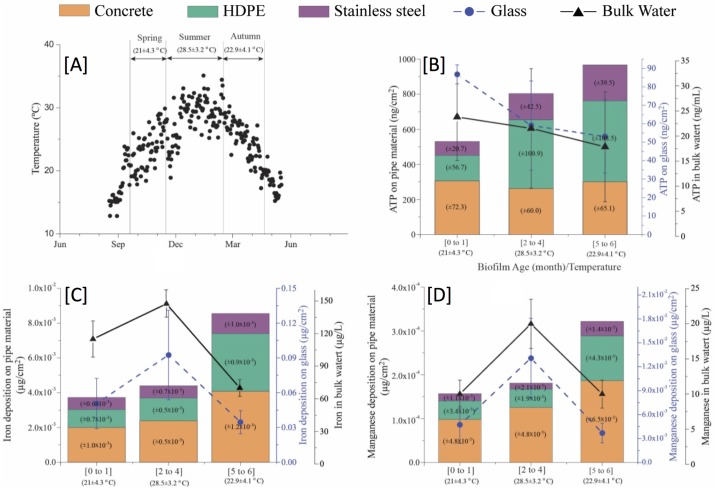
Details of monitored parameters during the study period. 2[A]: Bulk water temperature profile of entire study period. 2[B]: The live biomass (measured as a concentration of ATP) attached to different material and suspended in bulk water. 2[C]: The iron deposited on material and suspended in bulk water. 2[D]: The manganese deposited on material and suspended in bulk water. (± denotes the standard deviation)

The G&AWSS in Western Australia consists of a network of pipes that runs mostly aboveground. Since the pipes are aboveground they are exposed to the environmental elements (e.g. wind, rain, sun) and seasonal temperature variations of the atmosphere cause bulk water temperatures to change (Fig 2[A]). The study was carried out over Southern Hemisphere Spring (end of Oct to Nov), Summer (Dec to Feb) and Autumn (Mar to end of Apr) and the average bulk water temperatures for the respective seasons were 21.5 ± 4.3°C, 28.5 ± 3.2°C and 22.9 ± 4.1°C.

#### Plumbing material has an influence on biofilm formation

According to this study the ranking of the pipe material in the reduction of average ATP is as follows:

Stainless steel = Concrete < HDPE

During the first two months, a higher ATP was noted on concrete as apposed to on HDPE (Fig 2B). However, as the period of exposure increased the ATP on HDPE increased. This suggests that although the overall level of biofilm formation on concrete is lower than that of HDPE, the initial increase of biofilm formation on concrete is higher. Whether the slow increase of biofilm formation on HDPE and on stainless steel is a result of surface characteristics remains unclear. As the biofilm aged, the increase of ATP reduced on stainless steel, concrete and HDPE with no significant difference (p > 0.05) observed between concrete and stainless steel and a significant difference (p < 0.05) observed between HDPE and all other pipe materials (S1 Table).

#### Plumbing material and biofilm influence metal deposition

Biofilms can actively and passively contribute towards deposition of iron and manganese on pipe materials in drinking water systems [7, 45]. In the present study, iron and manganese deposition showed a similar trend to ATP on pipe material of MRD (Fig 2B, 2C & 2D). HDPE and stainless steel showed positive correlations between ATP and the deposited iron and manganese (Table 1). However, no correlations were observed with concrete. According to Tsai et al. [45], the positive correlation observed with HDPE and stainless steel is a result of colloidal iron and manganese oxides (deposited on plumbing material) enhancing attachment of bacteria. However, with stainless steel and HDPE being largely chemically inert, the deposition of iron and manganese may have been triggered upon an initial colonisation of bacteria on these two plumbing materials. The lack of correlation with concrete on the other hand is a result of the inherent ability of concrete to facilitate chemical deposition of iron and manganese [46, 47]. In this instance, chemical deposition has far exceeded biologically mediated deposition of iron and manganese on concrete. In summary, the plumbing material appears to influence (direct/indirect chemical and/or biological) surface deposition of metals in drinking water distribution systems.

**Table 1:** Relationship between ATP and deposited metals on pipe material.

Pipe material	R^2^ values for the regression line between ATP and deposited metals	
	Iron	Manganese
Concrete	0.0612	0.0135
HDPE	0.9017	0.8978
Stainless steel	0.9644	0.9342

Overall the ranking of the pipe material in terms of susceptibility to metals deposition (iron and manganese, determined based on the mean concentration of metals) is as follows:

Stainless steel < HDPE= Concrete 

According to mean deposition of iron and manganese, although concrete appears to be susceptible towards metals deposition, no statistically significant difference (p > 0.05) was observed between concrete and HDPE both at an early age and at a mature age of biofilm (S1 Table).

Overall this study was restricted to a single location of distribution system and since many factors (some of which are area dependent) determine the fouling (biological and chemical) potential (risk) of plumbing materials, further research is required to validate (e.g. under different hydraulic, environmental, disinfection and water quality conditions) observations made in this study.

#### Does the KIWA monitor in this mode of operation reflect biological fouling of MRD?

The roughened glass of the KIWA monitor did not mirror the increasing trends of ATP on coupons of the MRD (Fig 2B). Eboigbodin et al. [48] highlights the importance of keeping factors such as biofilm age, the microbial community, temperature and water flow velocities a constant when performing comparative studies of this nature. The KIWA monitor was operated as an offline device to the MRD (Fig 1) and although the biofilm on the roughened glass was of the same age and was exposed to the same microbial communities of the G&AWSS, the water flow velocities and the temperatures these biofilms were exposed to were different. Considering the device was exposed to reduce flow velocities (0.28 L min^-1^) and was more vulnerable to external elements, the difference perhaps is not surprising. It is also worth noting that the ATP measured on roughened glass in the KIWA monitor was on average remarkably lower (80%) than that recorded on the coupons of the MRD (Fig 2B). Further, a significant drop of ATP (Fig 2B) was noted over time (between 0 to 1 month and 2 to 4 months). With ATP measurements only reflecting the active biomass fraction, it is unclear whether the dead biomass fraction on roughened glass increased with time and biofouling still resembled coupons of MRD. Hence, KIWA monitors may necessarily not reflect an inaccurate measure of biofouling of drinking water distribution systems even when roughened glass of KIWA monitor and coupons of the MRD show no positive correlation for ATP.

With respect to active biomass fraction, significant differences (p < 0.05) were observed between glass of the KIWA monitor and the pipe materials of MRD (S1 Table). While other operational strategies (e.g. up flow instead of down flow, use of similar pipe material to that of distribution system) need exploration to enable a similar resemblance of active biomass, measurements other than ATP is required (e.g. extracellular polysaccharides, dead bacterial cell counts) to enable an accurate comparison of biofouling between coupons of MRD and rings of KIWA monitor.

#### Can the KIWA monitor be used to reflect chemical fouling of MRD?

Compared to the coupons on MRD, metal deposition on roughened glass of the KIWA monitor was substantially higher (Fig 2C & 2D). Specifically iron deposition on glass was 21 times higher than on concrete, 31 times higher than on HDPE and 70 times higher than on stainless steel. Similarly manganese deposition was 52 times higher than on concrete, 115 times higher than on HDPE and 343 times higher than on stainless steel. Accordingly, glass of the KIWA monitor showed statistically significant differences (p < 0.05) to pipe materials of MRD (S1 Table). Adsorption of cations, onto glass has been extensively studied [49] and stronger binding was observed with bivalent and trivalent cations (e.g. Cu^2+^ and Fe^3+^). Moreover, the lower water velocity (Reynold’s number ~ 300) maintained in the KIWA monitor compared to MRD (Reynold’s number ranging 0 to 34,000) may have resulted in lower shear forces, enabling glass rings to retain higher deposits of metal. Accordingly, the presence of excessive quantities of iron and manganese on roughened glass of the KIWA monitor is not surprising. Similar to glass, concrete and HDPE adsorb cations, and if the KIWA monitor is to reflect metal deposition on pipe material, the glass monitoring rings are best replaced with the same pipe material of the distribution system. While this change may contribute towards a positive outcome, other operational strategies may also need to be explored to successfully retrofit the use of the KIWA monitor to quantitatively estimate iron and manganese build up in distribution systems.

#### Whether bulk water could be used to make predictions remains inconclusive

In this study, the biofilms on coupons of MRD continued to mature during the experimental period. The gradual increases of total ATP on coupons of MRD support this claim (Fig 2B). The main distribution pipeline upstream of MRD, however, contained a mature biofilm and bulk water characteristics monitored reflect changes taking place in the larger distribution system. Hence, making an attempt to correlate bulk water changes to changes occurring on an immature biofilm on coupons of MRD is unrealistic.

Bulk water iron and manganese (Fig 2C & 2D) were highest during summer (147.5 ± 100 and 20 ± 0.3 μg L^-1^ respectively) and were less during spring (115 ± 70 and 10 ± 2 μg L^-1^ respectively) and autumn (70 ± 35 and 10 ± 2 μg L^-1^ respectively). High water flow velocities during summer [50] may have resulted in this observation. The higher demand for drinking water during summer causes an increase in the water flow rates through pipes, disturbing sediments at the bottom and/or on pipe surfaces leading to increases of iron and manganese concentrations in bulk water. However, the increase of bulk water iron and manganese during summer did not coincide with a release of deposited metal from coupons of MRD.

### Bacterial community compositions in bulk water, pipe material and KIWA monitor

454-pyrosequencing was performed to examine the bacterial community composition and diversity in bulk water, different pipe materials and on KIWA monitor. A total number of 83,829 sequences were generated from 14 different samples (bulk water, glass and pipe materials) and after processing the sequences (removing short sequences, denoising the sequences and filtering out chimeras) 75,668 sequences (nearly 90% of the total sequences) were used to examine bacterial diversity. The rarefaction curves generated at 97% sequence similarity (S1 Fig) highlight that a large percentage of the bacterial communities in each of the samples have been captured.

The bacterial diversity of the collected samples spread across a total 21 bacterial phyla. However, of the total sequences, ~78% belonged to phyla Proteobacteria (23.4 to 63.3%), Bacteroidetes (0.95 to 37.3%), Planctomycetes (3.1 to 22.2%), Armatimonadetes (5.8 to 19.6%), and Cyanobacteria (0.20 to 16.20%) (S2 Fig). With Proteobacteria outnumbering other bacterial phyla in all samples examined, this study together with other studies [51–55] confirm the dominance of Proteobacteria in drinking water distribution systems. Other bacterial phyla such as Chloroflexi, Gemmatimonadetes, Actinobacteria, Firmicutes, Verrucomicrobia, Acidobacteria were observed, but their abundance was only 3.0 to 11.2%. These bacterial phyla also have been noted in drinking water environments, and factors such as the source water quality (pH, organic matter, nitrogen etc.), the treatment process, the type of disinfectant residual maintained and water temperatures appear to influence their abundance in drinking water [56, 57].

#### Physicochemical parameters determine the extent of bacterial diversity

OTUs (defined based on 97% sequences similarity) varied between 207 to 523 (Table 2, determined based on the lowest sequences read of 2118) in the bulk water, glass and pipe materials. Irrespective of the type of media that facilitated biofilm growth, bacterial species richness (Chao1), phylogenetic diversity (PD) and bacterial diversity (Shannon index) were highest in bulk water (Table 2).

**Table 2:** Bacterial diversity indices of biofilms on pipe materials, glass and in bulk water.

Biofilm age & Temperature	Sample	Total Sequences	OTUs	OTUs*	Cho1	Shannon	PD	Comment
5–6 months 22.9 ± 4.1°C	Steel	5826	310	207	336.24	5.04	17.08	Indices decreasing with increasing biofilm age
	Concrete	6350	340	227	355.02	4.87	19.97	
	HDPE	8440	402	231	404.23	5.05	19.09	
2–4 months 28.5 ± 3.2°C	Steel	4113	346	271	416.98	6.51	25.88	
	Concrete	2246	310	310	417.11	6.66	28.68	
	HDPE	3596	395	319	506.9	6.47	27.2	
0–1 months (21.5 ± 4.3°C)	Steel	6275	398	273	422.28	6.43	25.78	
	HDPE	3730	356	294	438.84	6.41	27.07	
	Concrete	3843	392	313	520.14	6.67	28.01	
5–6 months 22.9 ± 4.1°C	Glass	7302	610	367	648.69	6.58	32.31	Indices increasing with increasing biofilm age
2–4 months 28.5 ± 3.2°C		2118	277	253	408.11	6.86	25.89	
22.9 ± 4.1°C (Autumn)	Bulk water	5390	776	523	893.18	7.36	44.91	Indices increasing with season
28.5 ± 3.2°C (Summer)		8519	845	473	789.44	7.31	40.25	
21.5 ± 4.3°C (Spring)		7920	750	443	765.35	7.15	37.78	

OTUs* determined based on minimum number 2118 observed in Glass.

Chao1, Shannon and PD were determined based on the lowest sequences number.

As a complete contrast to our study, Douterelo et al., [51] reported the bacterial diversity and species richness to be highest in biofilm (on HDPE pipe materials). Compared to the low chloramine concentration (total chlorine < 0.2 mg L^-1^) maintained in our study, the free chlorine residual (0.23 to 0.28 mg L^-1^) maintained in bulk water of the HDPE pipe network may have suppressed bacteria not capable of resisting disinfection residuals of 0.28 mg L^-1^, resulting in the observation made by Douterelo et al., [51]. Srinivasan et al. [58] also observed an increase of pipe wall biofilm and a decrease of bulk water bacteria when chlorine concentrations in bulk water was increased. This highlights the influence of the disinfection residual towards abundance and diversity of bacteria in bulk water and in biofilm. While past research could provide some insight to the observed differences of Douterelo et al., [51] and this study, an explanation that is indisputable can only be derived through a comparative study where all parameters (e.g. biofilm age, temperature water flow velocities etc.) except the residual chlorine is kept constant.

#### Diversity variations are based on biofilm age and not on colonization material

As the biofilm on pipe material aged, the bacterial diversity indices (Cho1, Shannon and PD) decreased (Table 2) and a notable difference was evident between ages 0 to 1 month and 5 to 6 month. However, an opposite trend was observed in biofilm of KIWA monitor. Liu et al. [59] reports a higher bacterial diversity in loose deposits (particulate matter residing at bottom of pipes) of distribution systems. In addition, an elemental analysis has revealed significant concentrations of aluminium, calcium, iron, magnesium, manganese, and arsenic like metals in loose deposits [59]. Our study revealed higher deposition of metals on roughened glass of the KIWA monitor and characteristics of glass was assumed to have contributed towards this observation. Accordingly, it is unclear whether higher metal concentrations contributed towards the higher bacterial diversity on roughened glass of the KIWA monitor.

**Fig 3:**
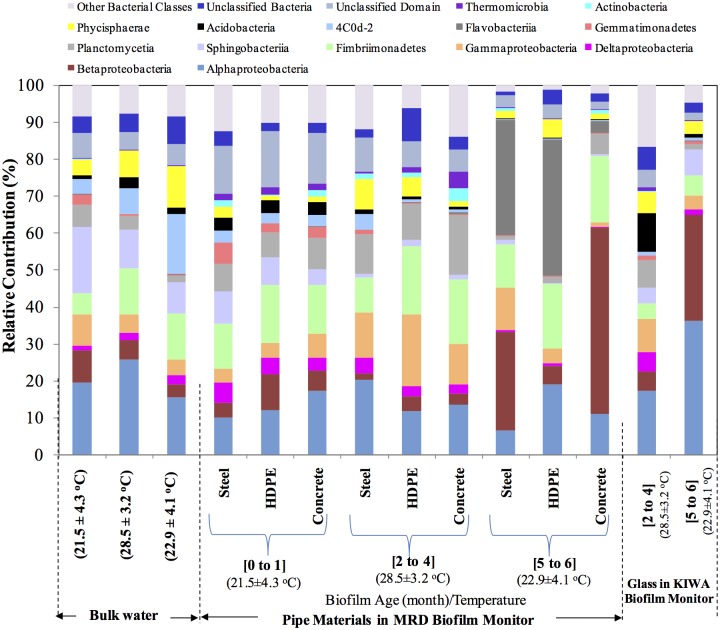
Comparison of the relative abundances of major bacterial classes found in biofilms on pipe materials and bulk water during three different sampling periods. Bacterial classes denoted on figure legend represents an abundance >3% in any of the samples. The legend “Other bacterial classes” refer to a total representation of bacterial classes having an abundance <3% in any given sample.

**Fig 4:**
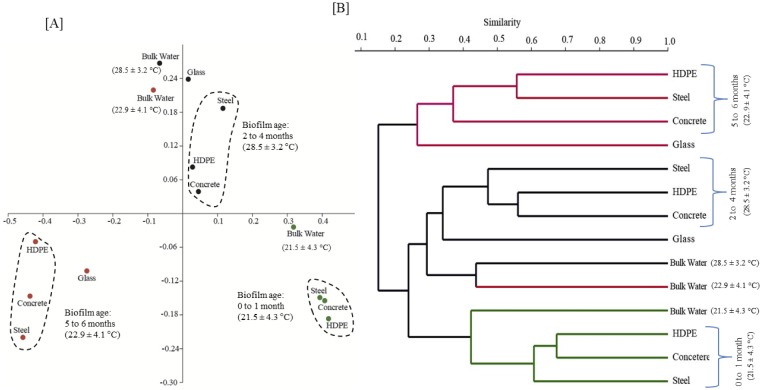
A similarity analysis (at 97% sequences similarity) of bulk water and biofilm samples (obtained from MRD & KIWA) based on Bray-Curtis similarity index: [A] Principal coordinates analysis (PCoA); [B] Cluster analysis.

The pipe material did not significantly contribute towards bacterial diversity differences in pipe wall biofilms (Table 2, Fig 3). When a PCoA based on 97% sequence similarity was carried out, bacterial communities were found to differ based on biofilm age and not on colonisation material (Fig 4A). However, according to the cluster analysis (Fig 4B), some differences were noted amongst biofilms on different pipe materials of similar age. For example, only an overall 55% similarity was observed across biofilms of all pipe materials when age of biofilm was 0 to 1 month. Between biofilms of HDPE and concrete, a higher similarity (68%) was observed at this early age of biofilm. As the biofilm aged, the differences between biofilms of pipe materials widened with the overall similarity decreased to 47% at a biofilm age of 2 to 4 months and to 36% at an age of 5 to 6 months (Fig 4B).

The above observation is further consolidated when bacterial phyla were compared on pipe materials at different ages of biofilms. The abundance of some bacterial phyla increased while some decreased with increasing age of biofilm. For example: at a biofilm age of 0 to 1, Proteobacteria abundance on steel and on concrete was 23% and 33% respectively (S2 Fig). On reaching an age of 5 to 6 months, the abundance increased to 46% and 63% respectively. However, with phylum Acidobacteria the abundance (5% on steel and 4% concrete) decreased with the age of biofilm (0.8% on steel and 0.7% concrete) (S2 Fig). The differences in abundance were found to be more pronounced at a genus level (S2 Table).

Biofilm management in a distribution system is reliant on a disinfectant residual that is effective towards the bacterial diversity on pipe wall biofilms [60]. Fig 3 demonstrates a relatively uniform bacterial community across all pipe materials specifically during early age of biofilm. For example during the age of 0 to 1 month, the variation of relative abundance (expressed as a %) between pipe materials for class Gammaproteobacteria was ±1.5. The variation increased with age to ±4.6 and to ±5.3 suggesting each material is becoming selective towards specific bacterial communities. Future research could explore whether higher phylogenetic similarity of young biofilms on pipe materials (Fig 4A), could be exploited to better manage disinfection residuals of distribution systems having concrete, HDPE, stainless steel or a mixture of the above pipe materials.

#### Monitoring bulk water could only reveal 40% of bacterial diversity of MRD

The phylogenetic similarity between bulk water bacteria and biofilms of pipe materials decreased as the biofilms aged (Figs 3 and 4). Specifically an overall 42% similarity was observed when the age of biofilm was 0 to 1 month. Similarity further decreased down to 29% when the age of biofilm was 2 to 4 months and once an age of 5 to 6 months was achieved only a 15% similarity was observed (Fig 4B). Accordingly, if a pipe wall biofilm is young (e.g. 0 to 1 month), bulk water bacterial communities could be used to describe a larger fraction of the biofilm’s bacterial phylogeny.

**Fig 5:**
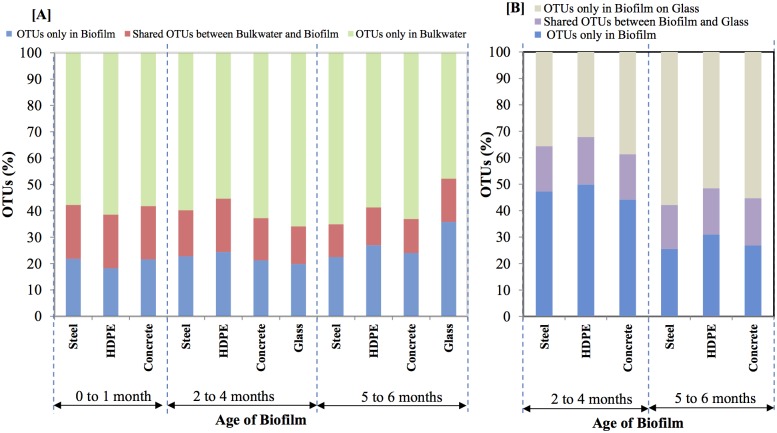
Details of shared and unique bacterial OTUs between [A] bulk water and pipe materials of MRD and KIWA biofilm monitor [B] pipe materials of MRD and KIWA biofilm monitor.

An examination of shared OTUs (as a % of total OTUs observed on each pipe material and bulk water) between bulk water and pipe materials and singleton OTUs in bulk water and in biofilms on pipe materials (Fig 5A) further consolidated the above observation. In summary, a higher number of shared OTUs (>20% of the total OTUs) were observed with a young pipe wall biofilm (Fig 5A). Some of the bacterial genera observed among shared OTUs included *Rubrivivax* of family Comamonadaceae and *Planctomyces* of family Planctomycetaceae (S2 Table). Consistent with the cluster analysis, the shared OTUs gradually decreased with increasing biofilm age of pipe materials (Fig 5A).

#### Could KIWA monitors reflect the bacterial diversity of MRD?

When similar age biofilms (e.g. biofilm ages of 2 to 4 and 5 to 6 months) were compared, only a 33% similarity was observed between biofilms of KIWA monitor and MRD (Fig 4B). An examination of shared OTUs (as a % of total OTUs observed on each pipe material and glass of KIWA) between KIWA and pipe materials and singleton OTUs in biofilms on KIWA and on pipe materials (Fig 5B) further consolidated the above observation (i.e. there was only 10% of shared OTUs). As the biofilms on KIWA and pipe materials aged, the similarity between the two decreased further (Fig 4B). This further suggests that pipe materials have an influence on the bacterial diversity of a biofilm. Specifically pipe composition, age, surface roughness was found to impact biomass deposition on pipe material [19, 61]. Based on this understanding, van der Kooij et al. [32] used inert glass and Teflon in KIWA monitors to explore whether offline biofilm monitoring could be used to express water quality (in terms of biofilm formation rate) and biofilm formation potential (in terms of maximum level of biomass accumulation) in drinking water distribution systems. The inert glass and Teflon surfaces were specifically chosen to remove the effects of pipe materials on biomass accumulation, (e.g. with a release of biodegradable compounds or compounds inhibiting growth) allowing bulk water characteristics to be the only factor governing biofilm formation in the KIWA monitors. However, as the use of glass in KIWA monitors failed to phylogenetically reflect bacterial diversities in both the bulk water and pipe material (Figs 3B and 5B), whether the use of a KIWA monitor (fitted with inert glass) could be extended to reflect microbial quality of bulk water remains questionable. Further, the ATP measured on roughened glass in the KIWA monitor was 80% less compared to that on the coupons of the MRD (Fig 2B). The KIWA monitor in this mode of operation (detailed in this study) was unable to reflect the biofilm formation potential of bulk water in the G&AWSS.

## Conclusions

In this study, a MRD was installed in the G&AWSS in Western Australia to investigate biofouling and iron and manganese deposition on different pipe materials (concrete, HDPE and stainless steel). To examine whether pipe wall biofilms could be monitored through alternative means, offline biofilm (a KIWA monitor) and bulk water monitoring were employed. Biofouling was examined at community level using 454 pyrosequencing. The findings of the study can be summarized as follows:

- HDPE pipe surfaces were more susceptible towards biofouling while concrete surfaces were towards metals deposition.
- KIWA monitors under operational conditions used in this study do not reflect biological and chemical fouling of pipe surfaces. Roughened glass of KIWA influenced abiotic deposition of metals.
- Bacterial diversity and phylogenetic similarity decreased with age of biofilm on concrete, HDPE and stainless steel pipe surfaces. However, bacterial diversity increased on roughened glass of KIWA monitor.
- Bulk water revealed 40% of bacterial diversity found in young pipe wall biofilms.

## Supporting Information

S1 Table Statistical analysis of chemical and biofouling on different pipe materials using one way ANOVA. (TIFF)

S2 Table Bacterial community composition at genus level observed in biofilms on pipe materials, glass and bulk water at different ages of biofilm and bulk water temperature. (TIFF)

S1 Fig Rarefaction curves of OTUs in the biofilm of different pipe materials, glass and bulkwater at different age of biofilm and bulkwater temperature at 97% sequences similarity. (TIFF)

S2 Fig Comparison of the relative abundances of major bacterial phyla at different age of biofilm and bulk water temperatures on pipe materials, bulk water and KIWA monitor. Bacterial phyla denoted on figure legend represents an abundance >3% in any of the samples. The legend “Other phyla” refer to a total representation of bacterial phyla having an abundance <3% in any given sample.
(TIFF)
