# Supplementary figures and images for: Effectiveness of Devices to Monitor Biofouling and Metals Deposition on Plumbing Materials Exposed to a Full-Scale Drinking Water Distribution System
Source: PLoS One. 2017 Jan 6;12(1):e0169140. doi: 10.1371/journal.pone.0169140 (PMC5218461; doi:10.1371/journal.pone.0169140)

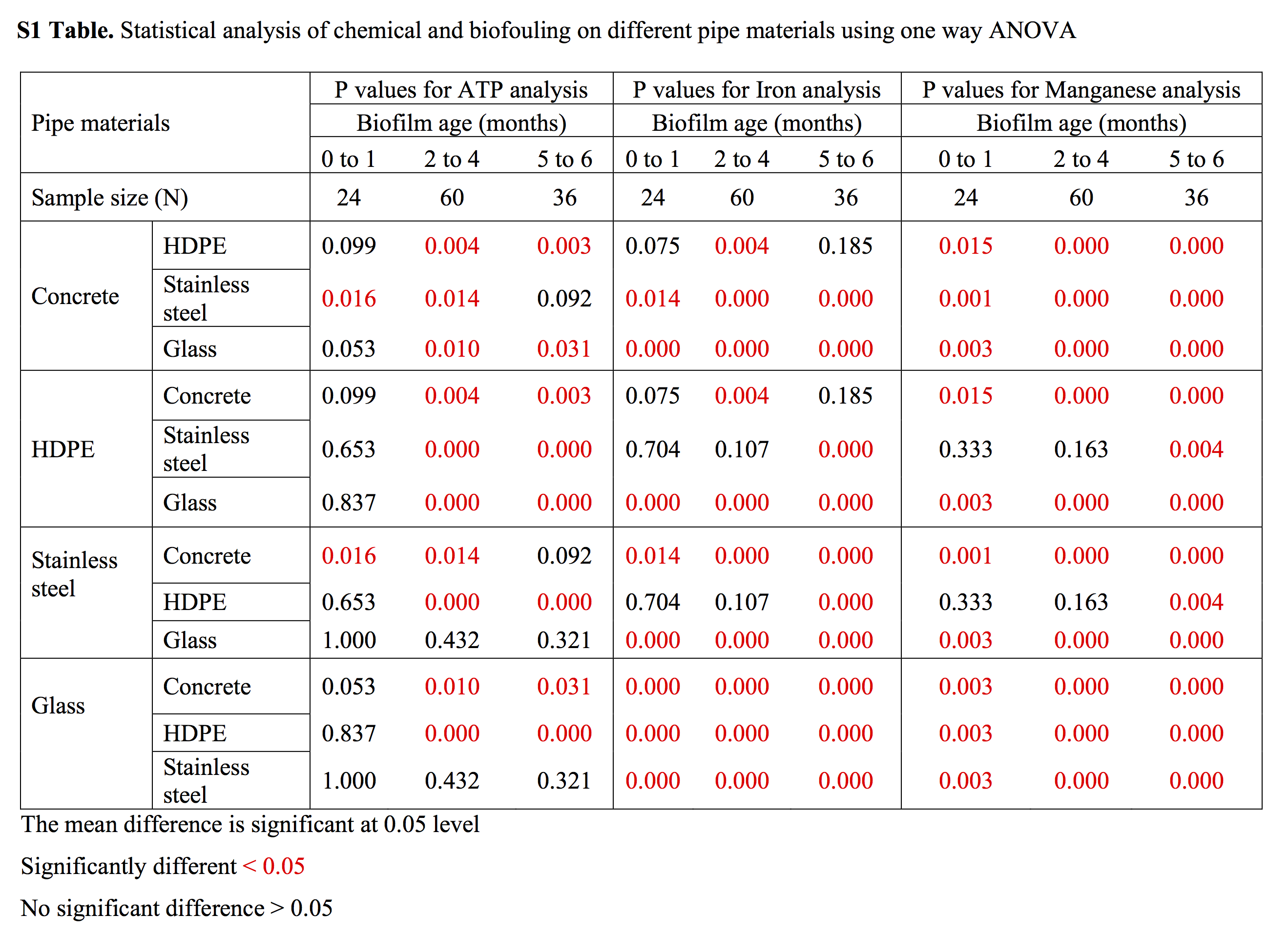

Supplement: S1 Table — (TIFF) [file pone.0169140.s001.tiff]

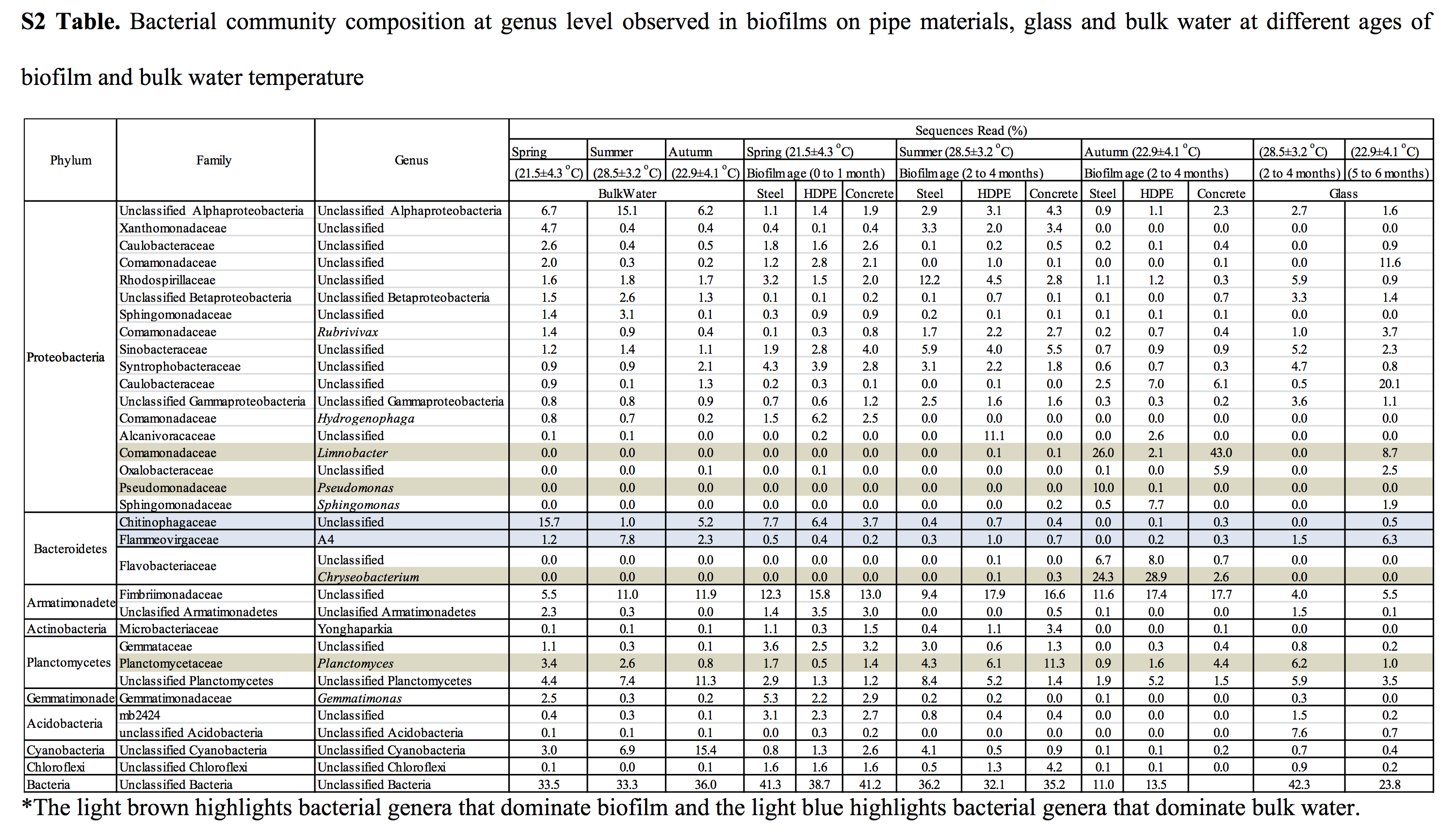

Supplement: S2 Table — (TIFF) [file pone.0169140.s002.tiff]

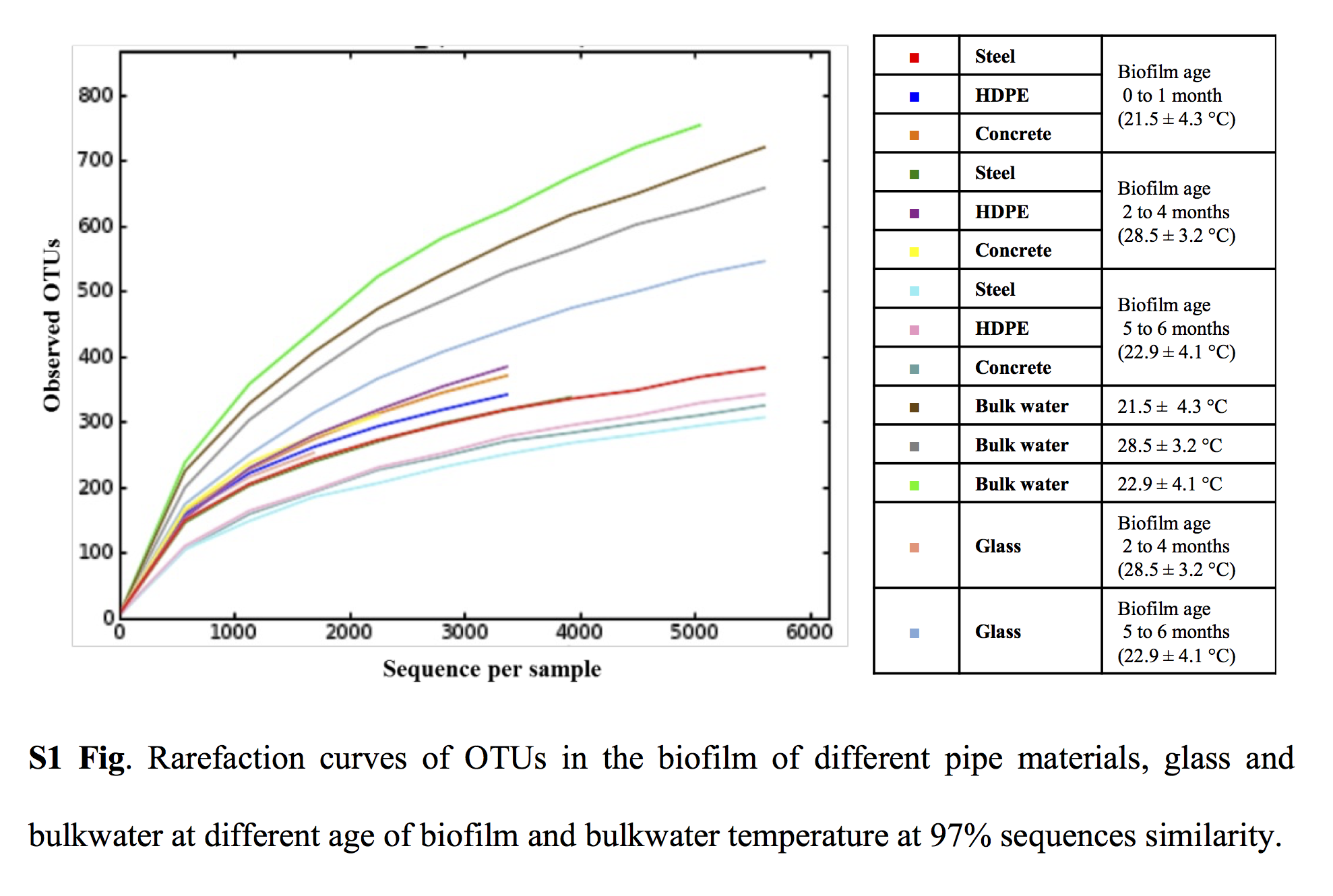

Supplement: S1 Fig — (TIFF) [file pone.0169140.s003.tiff]

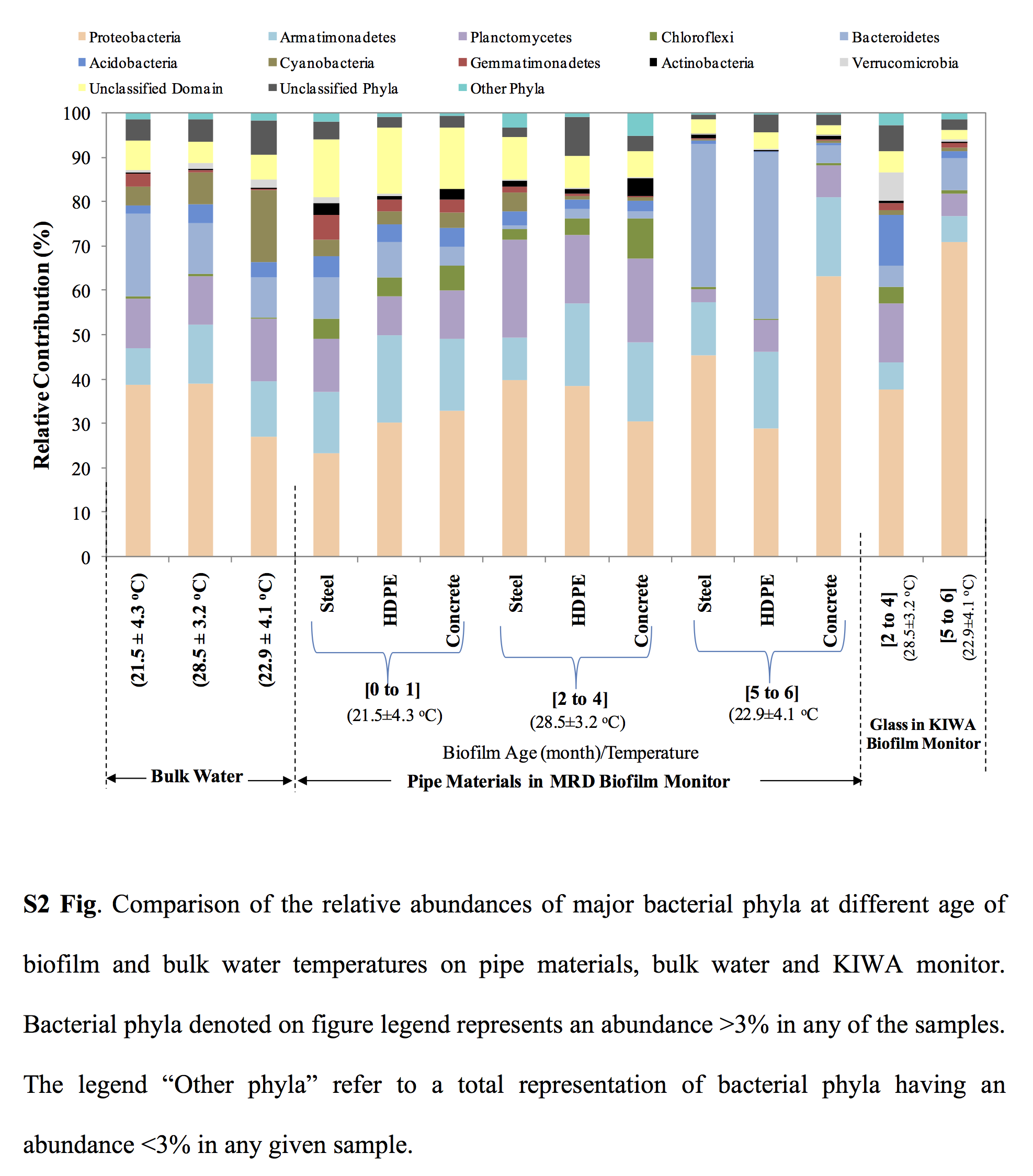

Supplement: S2 Fig — Bacterial phyla denoted on figure legend represents an abundance >3% in any of the samples. The legend “Other phyla” refer to a total representation of bacterial phyla having an abundance <3% in any given sample. (TIFF) [file pone.0169140.s004.tiff]
